# Nabiximols is Efficient as Add-On Treatment for Patients with Multiple Sclerosis Spasticity Refractory to Standard Treatment: A Systematic Review and Meta-Analysis of Randomised Clinical Trials

**DOI:** 10.2174/1570159X21666230727094431

**Published:** 2023-09-25

**Authors:** Dénes Kleiner, István László Horváth, Stefania Bunduc, Dorottya Gergő, Katalin Lugosi, Péter Fehérvári, Péter Hegyi, Dezső Csupor

**Affiliations:** 1 University Pharmacy, Department of Pharmacy Administration, Semmelweis University, Hőgyes Endre utca 7-9., 1092 Budapest, Hungary;; 2 Centre for Translational Medicine, Semmelweis University, Üllői út 26, 1085 , Budapest, Hungary;; 3 Division of Pancreatic Diseases, Heart and Vascular Center, Semmelweis University, Baross út 22-24, 1085 Budapest, Hungary;; 4 Faculty of Medicine, Carol Davila University of Medicine and Pharmacy, Dionisie Lupu Street 37, 020021, Bucharest, Romania;; 5 Center of Digestive Disease and Liver Transplant, Fundeni Clinical Institute, Fundeni Street 258, 022328, Bucharest, Romania;; 6 Department of Pharmacognosy, Semmelweis University, Üllői út 26., 1085 Budapest, Hungary;; 7 Department of Neurology, Bajcsy-Zsilinszky Hospital, Maglódi Road 89-91, 1106 Budapest, Hungary;; 8 Budapest Department of Biostatistics, University of Veterinary Medicine, István utca 2., 1078 Budapest, Hungary;; 9 János Szentágothai Research Center, University of Pécs, Szigeti út 12, 7624 Pécs, Hungary;; 10 Institute for Translational Medicine, Medical School, University of Pécs, Szigeti út 12, 7624 Pécs, Hungary;; 11 Institute of Clinical Pharmacy, University of Szeged, Szikra utca 8, 6725 Szeged, Hungary;; 12 Department of Pharmacognosy, University of Szeged, Eötvös u. 6, 6720 Szeged, Hungary

**Keywords:** Nabiximols, THC/CBD, Sativex, multiple sclerosis, resistant spasticity, spasticity numerical rating scale, systematic review, meta-analysis

## Abstract

**Background:**

Spasticity affects 54% of multiple sclerosis (MS) patients at disease onset, but this rate gradually increases with disease progression. Spasticity does not fully respond to standard treatment in one-third of the patients.

**Objective:**

Our systematic review and meta-analysis assessed whether add-on nabiximols, can improve MS-associated refractory spasticity.

**Methods:**

The systematic literature search was performed in Web of Science, MEDLINE, Scopus, CENTRAL, and Embase, on 15/10/2021, without restrictions. We included in the review blinded, randomized, placebo-controlled trials evaluating the efficacy of nabiximols in adult MS patients with refractory spasticity, by comparison with placebo. The primary outcome was responder rate by spasticity numerical rating scale (NRS). Secondary outcomes were spasticity-related parameters. We used random effect models to calculate odds ratios (OR) or mean differences and the corresponding 95% CI. Bias-factors were assessed with Cochrane risk of bias tool (RoB2). (PROSPERO ID: CRD42021282177).

**Results:**

We identified 9 eligible articles, of which 7 (1128 patients) were included in the meta-analysis. The spasticity numerical rating scale (NRS) was significantly higher in the nabiximols group than in the placebo group (OR 2.41 (95% CI 1.39; 4.18)). Secondary outcomes were in accordance with our primary results. At least some concerns were detected in the risk of bias analysis.

**Conclusion:**

Our results indicate that nabiximols is efficient in MS associated spasticity, refractory to standard treatment and it may be considered as add-on symptomatic therapy. Nevertheless, further studies are needed to establish the optimal treatment protocol – dose, duration, moment of initiation, disease type.

## 1 INTRODUCTION

Multiple sclerosis (MS) is one of the most frequent disorders of the central nervous system with a general prevalence of 35.9/100,000 people worldwide [1]. Spasticity is a common symptom of MS [1], affecting around 54% of patients at disease onset, and up to 87-91% of patients 20 years after diagnosis [2]. Treatment-resistant spasticity affects one-third of the patients with MS [3]. In a German survey, physicians reported incomplete or lack of pharmacological treatment response in one-fifth of the 252 cases treated with antispastics, while for the severe forms this rate increased to 60% [4].

Spasticity is most commonly evaluated by the treating physician with the Ashworth Scale (AS), or its modified version (mAS). They measure the resistance against passive movement [5]. Although it is not a patient-reported outcome, the results may still be influenced by the rater’s subjectivity and it only measures one feature of spasticity [5]. At the same time, a much wider range of symptoms can be associated with spasticity, including muscle spasms, pain, sleep disturbances, fatigue, poor motor control, slowed movement, and impaired bladder function, among others [5]. The numerical rating scale (NRS) is a 0-10 self-reported parameter similar to the visual analog scale (VAS), where 0 means no spasticity and 10 means worst spasticity ever [5]. While subjectivity may raise concerns, assessing the subject’s own perception may be more appropriate for spasticity measurement considering its complex clinical implications [5, 6].

The effectiveness of cannabinoids in the spasticity of various causes has been investigated multiple times with contradicting results [7, 8]. Nabiximols (trade name: Sativex^®^) is an oromucosal spray containing a well-defined extract of *Cannabis sativa* L. (27 mg delta-9-tetrahydrocannabinol and 25 mg cannabidiol per ml) [9]. Although nabiximols is marketed as a medicine in several countries, like Austria, Australia, Great Britain, Germany, Italy, and Spain, it is typically just the last treatment option before intrathecal baclofen [10, 11].

The above-mentioned conflicting results raise the question of whether nabiximols are effective against MS-related refractory spasticity. Therefore, the aim of our systematic review and meta-analysis was to investigate whether nabiximols can improve the symptoms of MS-associated spasticity refractory to standard treatment as an add-on agent.

## 2 MATERIALS AND METHODS

### 2.1 Search Strategy

To ensure the quality of our systematic review and meta-analysis, Cochrane methodology recommendations and the Preferred Reporting Items for Systematic Reviews and Meta-Analyses (PRISMA) 2020 statement have been followed [12, 13]. We registered the protocol for our analysis to PROSPERO (International prospective register of systematic reviews) (CRD42021282177) and followed the protocol without deviations.

We followed the “PICO-S” (*i.e*. population, intervention, comparator, outcome, study design) approach for the formulation of the clinical question and defining the eligibility criteria. The population included adult patients (age ≥ 18 years), diagnosed with MS, receiving adequate disease-modifying and antispastic therapy, but with inadequate spasticity response. The intervention was nabiximols as an add-on treatment, while the comparator was a placebo as an add-on treatment. The primary outcome was the spasticity response rate. The secondary outcomes were the change of spasticity and spasticity-related parameters, such as spasticity NRS or VAS; mAS, timed 10-m walk, subject global impression of change (SGIC), sleep disturbance, Barthel Index for Activities of Daily Living (ADL) and quality of life. Regarding study design, only blinded, randomized, placebo-controlled trials were eligible for analysis. Any other study designs were deemed ineligible.

The search was performed within the Web of Science, MEDLINE (*via* PubMed), Scopus, Cochrane Central Register of Controlled Trials (CENTRAL), and Embase databases on the 15^th^ of October, 2021. The following search key was used: (*multiple sclerosis OR “disseminated sclerosis” OR “encephalomyelitis disseminata”*) *AND* (*nabiximols OR Sativex*); without filters or restrictions.

### 2.2 Selection

The selection was performed according to the above-mentioned eligibility criteria using the Endnote 20 (Clarivate Analytics, Philadelphia, PA, USA) software. Automatic and manual duplicate removal of the records were performed (DK). Two independent investigators (DK and ILH) performed the selection in two phases: based on the title and abstract, and subsequently on full-text contents. Cohen's kappa coefficient (k) was calculated to evaluate inter-rater agreement after each selection step. Significance levels are described in Table **S1a**. Disagreements were resolved by a third independent investigator at each step of the selection (DCs and SB). If the systematic search did not retrieve the trial protocol for the identified eligible studies, we searched them in https://clinicaltrials.gov/ and www.clinicaltrialsregister.eu registries. If the protocols were not published, we contacted the corresponding authors to check for their availability.

### 2.3 Data Collection

Data was extracted manually (DK) to a standardized Excel (Microsoft Corporation, Redmond, Washington, USA) sheet and checked by an independent investigator (DG). The following data were collected from each eligible article: publication details (authors, year of publication, country of origin, Digital Object Identifier (DOI)); study characteristics (study type, follow-up period, sample size); patients characteristics (sex distribution, age, disease type, and stage); information about the intervention (dose, route of administration and duration of treatment); information about the outcomes (spasticity response rate, spasticity level measured by NRS or VAS; Barthel ADL, timed 10-m walk, quality of life, the general impression of change, AS, mAS) as reported in each of the eligible articles.

We used the Plot Digitizer if data was available in graphical form only [14, 15]. In case of inconsistencies between the data reported in the published article and the data reported in the protocol registry, we extracted the data from the peer-reviewed publication.

### 2.4 Statisctial Analysis

For NRS and SGIC responder rates the raw data from the selected studies were pooled using random-effect models with the Mantel-Haenszel method [16, 17]. To be able to pool the results together, due to some measurement dissimilarities across the eligible studies (*i.e*. differing minimum, maximum, or distance, in the case of the 25 ft walking test), we had to recalculate some data. These are presented in the supplementary material (Tables **S1b-f**).

In the case of continuous variables pooling mean change differences (MD) necessitates the knowledge of the standard deviation (SD) of within-group difference between time points or the correlation of within-group changes, however, most studies reported neither. In these cases, we used the sum of the reported before and after treatment group SDs as a conservative (over) estimate of variability. The analysis of SGIC responders was assessed in accordance with Table **S1g**. Where possible we used the type of study design as an explanatory variable (*i.e*. subgroups) to account for the introduced heterogeneity. For all models, τ2 was estimated with the Paule-Mandel method [18], and the Q profile method for calculating the confidence interval of τ2 [19]. A funnel plot of the logarithm of effect size and comparison with the standard error for each trial was used to evaluate publication bias. Statistical heterogeneity across trials was assessed by means of the Cochrane Q test, and the I2 values [20]. I2 values of 25, 50, and 75% were identified as low, moderate, and high estimates, respectively. Outlier and influence analyses were carried out following the recommendations of Harrer *et al*. [19] and Viechtbauer and Cheung [21].

All analyses were conducted in R version 4.1 [22] using the following packages; tidyverse [23], meta [24], dmetar [19] metafor [25].

### 2.5 Risk of Bias Assessment

The assessment of bias factors of the publications included in our analysis was performed by 2 independent investigators (DK and SB) using the Cochrane risk of bias tool (RoB2) [26]. Five main domains were evaluated: randomization process, deviations from intended interventions, missing outcome data, measurement of the outcome, and selection of the reported results [26]. The overall risk of bias could be “Low”, “Some concerns” or “High”. Disagreements were resolved by consensus.

### 2.6 Certainty of Evidence Assessment

The certainty in the body of evidence was evaluated by the Grading of Recommendations, Assessment, Development and Evaluations (GRADE) framework (DK, checked by SB) [27, 28]. The level of evidence could be evaluated as “High”, “Moderate”, “Low” or “Very low”. Each outcome was rated on a 9-point scale as - “critical” (9-7); “important” (6-4); or “not important” (3-1).

## 3 RESULTS

### 3.1 Study Characteristics

We found 14 reports eligible for our review, including the registered trial protocols for 5 of the studies [29-33]. Nine articles (1510 patients) were included in the systematic review and 7 (1135 patients) provided data for meta-analysis [6, 34-39]. The selection process is detailed in Fig. (**1**).

The mean duration of MS before study enrolment was between 12.0 and 22.1 years. A common inclusion criterion across the eligible studies was minimum 4-week stability of the treatment and of the disease before recruitment. All but one eligible study recruited patients with at least moderate spasticity [40]. Although the study of Langford *et al*. (2013) investigated the effect of nabiximols on pain in MS patients [40], they also reported the results for spasticity response to nabiximols, therefore we included them in our systematic review. Study and patient characteristics are summarized in Tables **1** and **2**.

### 3.2 Primary Outcome

The results for the overall spasticity response rate and subgroup analyses based on the study design are summarized in Fig. (**2**). Spasticity response rate was significantly higher in the nabiximols group by comparison with the placebo group) OR 2.41; 95% CI 1.39-4.18; I^2^ = 68%; *p* = 0.01). In the study of Leocani *et al*. (2015) the cut-off point for the response was a 20% decrease in spasticity as evaluated by NRS, and the treatment period was only 4 weeks [38]. Moreover, this was the only cross-over study that could be included to this analysis. In the other studies, patients were treated for at least 6 weeks and the response cut-off point was at 30%. The effect was higher in the studies with enriched design than in the other studies [37, 39].

### 3.3 Secondary Outcomes

**Fig. (3a and b):**
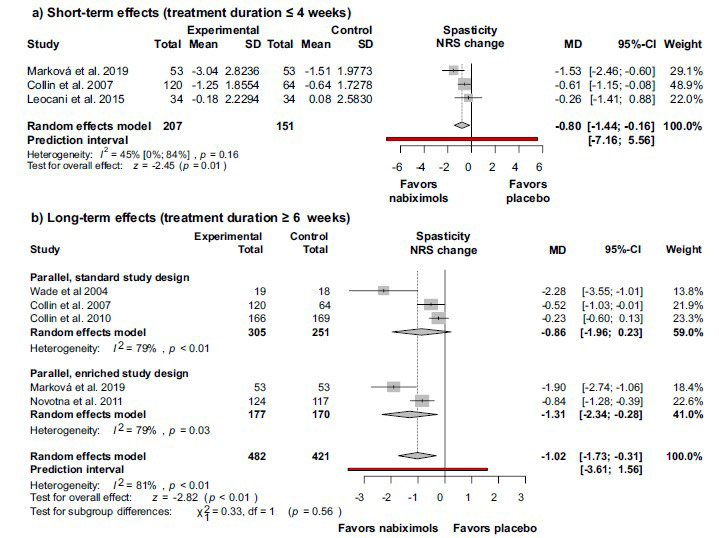
Nabiximols significantly alleviates the severity of multiple sclerosis associated spasticity as measured by numerical rating scale -both after a short-term (**a**) and a long-term (**b**) treatment - by comparison with placebo. Short term treatment’s duration was less than 4 weeks (**a**), long term treatments were administered for 6 weeks to 16 weeks (**b**). **Abbreviations**: Confidence interval (CI); mean difference (MD); numerical rating scale (NRS).

The efficacy of nabiximols indicated by the results for the primary outcome was further supported by the differences in the NRS values between the intervention and control groups (Figs. **3a** and **b**). Three articles reported on the short-term effects of nabiximols (treatment duration ≤ 4 weeks) [6, 38, 39], and 5 articles reported on the long-term effects of nabiximols (treatment duration ≥ 6 weeks) [6, 34, 36, 37, 39]. The decrease in spasticity was more prominent after long-term treatment, and in both cases, the difference between the intervention and the control group was statistically significant, yet of limited clinical relevance (for short-term treatment: MD -0.80; 95% CI (-1.44)-(-0.16); I^2^ = 45%; *p* = 0.16; for long term treatment: MD -1.02; 95% CI (-1.73) – (-0.31); I^2^ = 81%; *p <* 0.01).

**Fig. (4):**
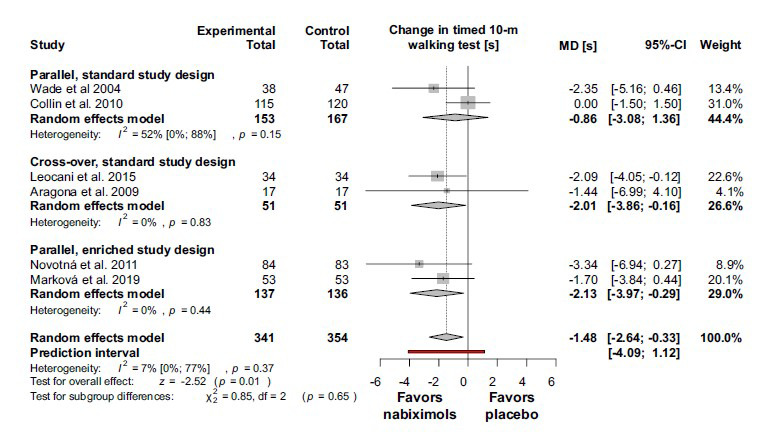
Nabiximols improve gait, measured by 10-m timed walking test by comparison with placebo in multiple sclerosis patients with spasticity refractory to standard treatment. **Abbreviations**: Confidence interval (CI); mean difference (MD); seconds (s).

In accordance with the results for the primary outcome, the 10-m timed walk was improved by nabiximols. The mean difference by comparison with placebo was -1.48 (95% CI (-2.64) –(-0.33); I^2^ = 7%; *p* = 0.37) (Fig. **4**).

**Fig. (5):**
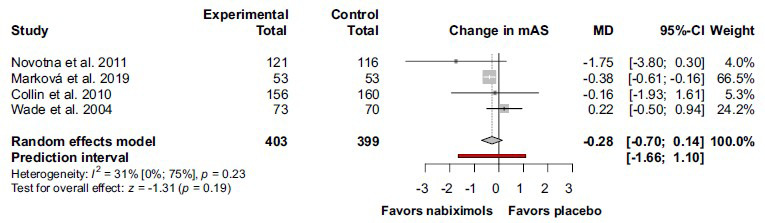
There was a tendency for improvement in the modified Ashworth scale in patients with multiple sclerosis, treated with nabiximols by comparison with placebo. **Abbreviations**: Confidence interval (CI); modified Ashworth scale (mAS); mean difference (MD).

On the other hand, in the case of mAS the change was not statistically significant (MD -0.28; 95% CI (-0.70) –0.14; I^2^ = 31%; *p* = 0.23) (Fig. **5**).

**Fig. (6):**
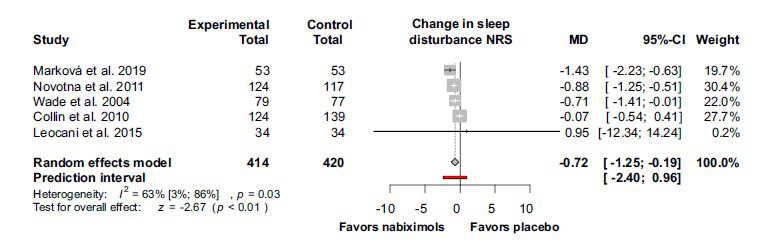
Sleep disturbance was ameliorated by nabiximols in patients with multiple sclerosis, treated with nabiximols by comparison with placebo. **Abbreviations**: Confidence interval (CI); mean difference (MD); numerical rating scale (NRS).

The effect on sleep disturbance was calculated based on 5 studies and revealed a significant beneficial effect of nabiximols (MD -0.72; 95% CI (-1.25) –(-0.19); I^2^ = 63%; *p* = 0.03) (Fig. **6**).

**Fig. (7):**
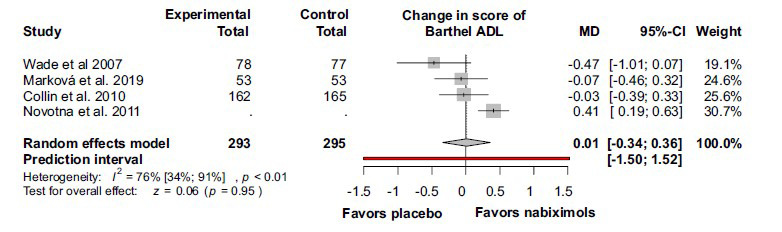
There was no significant difference between the nabiximols and the placebo groups in Barthel ADL in patients with multiple sclerosis. **Abbreviations**: Activities of daily living (ADL); confidence interval (CI); mean difference (MD); numerical rating scale (NRS).

The Barthel ADL scores also did not differ significantly between the groups (MD 0.01; 95% CI (-0.34) –0.36; I^2^ = 76%, *p <* 0.01) (Fig. **7**).

**Fig. (8):**
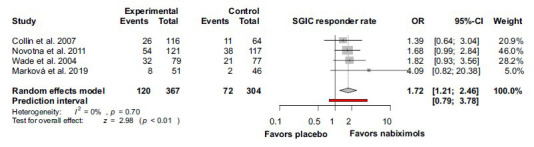
The SGIC was significantly improved in the nabiximols group compared to placebo group with multiple sclerosis. **Abbreviations**: Confidence interval (CI); mean difference (MD); subject global impression of change (SGIC).

Nabiximols showed a significantly higher response rate in the case of SGIC as well, when compared to placebo [OR 1.72; 95% CI 1.21-2.46; I^2^ = 0%; *p* = 0.70) (Fig. **8**).

Because of the differing baseline characteristics of the patients in the study of Novotna *et al*. (2011) we performed additional analyses by excluding their data for all the corresponding outcomes and obtained similar results [37] (Figs. **S1-S5**).

### 3.4 Qualitative Synthesis

Some of the eligible studies reported data we could not include in our meta-analyses due to a lack of data dispersion reporting while the AS was reported in only 2 studies. From the collected spasticity NRS, AS, and mAS we have found no further statistically significant differences.

Quality of life parameters were reported in 5 studies (1055 patients) (Table **S2**). Due to the differing measurement scales and the lack of data dispersion reporting we could not perform a meta-analysis for this outcome. Results were mixed, and the difference between the groups was not significant. The two exceptions were the SF-36 social functioning domain in Langford *et al*. (2013) [40], when placebo led to significantly better results than nabiximols, and SF-36, bodily pain domain in Marková *et al*. (2019) [39], when nabiximols performed better.

### 3.5 Risk of Bias Assessment and Quality of Evidence

In our review, we could not find any article with a low overall bias risk. Most frequently data about allocation sequence concealment was missing. Moreover, there were discrepancies between the peer-reviewed published data and the data reported in the trial protocol registries regarding the number of participants and the results (Figs. **S6a-h** and **S7**).

The level of evidence for our findings varied from moderate to low and very low. Besides the often high risk of bias we identified in the eligible studies, other reasons for downgrading were inconsistency, indirectness, and imprecision (results available in Table **S3**). The level of evidence was moderate for the results on spasticity responder rate and the alleviation of spasticity NRS in the long-term treatment subgroup, low for the short-term treatment subgroup alleviation of spasticity NRS, and very low for all other parameters. Publication bias was not evaluated, because of the low number of available articles.

## 4 DISCUSSION

The therapy of MS-related spasticity relies on the use of baclofen, tizanidine, and to lesser extent gabapentin, dantrolene and benzodiazepines [11]. In countries, where nabiximols are already accepted in the treatment of MS-related resistant spasticity the guidelines recommend switching between symptomatic treatment lines in case of no clinical response, meanwhile, the definition of inefficiency is not enough detailed and can slow down the add-on of nabiximols [11]. Moreover, it is not surprising that in countries without acceptance it is still a question whether nabiximols are effective – or not, even if it is a widely confirmed treatment option [11, 42]. It is also unclear how nabiximols work against spasticity. Mechanisms proposed include modulation of glutaminergic and endocannabinoid transmission [43].

In our meta-analysis, we confirmed the efficacy of nabiximols as an add-on agent in the symptomatic management of MS-associated, treatment-resistant spasticity. This improvement can be confirmed by the further ease of other spasticity-associated symptoms including - gait-control, sleep disturbance, and a global feeling of improvement.

For the primary outcome, a notable point should be highlighted. In the analysis of spasticity NRS responder rates, we performed our meta-analysis with the inclusion of a 4-week-long study [38]. As it has been mentioned, at least a 20% reduction in the NRS score was the cut-off point for responders in this study, meanwhile, a 30% decrease is accepted in every other included article. This difference presumably did not distort the results, because it has been already proven by Collin *et al*. (2010) that patients [36], who achieve a 20% decrease in the first 4 weeks, have a high chance to reach the threshold of 30% in the further period. On the other hand, around 63% of patients would respond to a nabiximols treatment. This correlates well with a report on the Italian multicenter dataset, the SA.FE (SAtivex efFEcts) study [44]. From the 1615 enrolled patients, 1010 (*i.e*. 63%) achieved a 20% decrease after one month of nabiximols treatment, with no respect to drop-out.

In accordance with the responder rate, the level of spasticity as evaluated by the NRS, significantly improved in short-term and long-term treatments as well. While generally a unit change can be expected, in practice the outcomes maybe even better. This scenario is best modelled by the enriched study design protocols when essential non-responders are excluded, and patients may achieve a spasticity NRS around 4.1-5.8 from an initial scale of 5.4-7.1 by our results. This decrease of 18-24% is in line with the results of the observational studies from the last decade. In the German MOVE (Mobility Improvement (MObilitätsVErbesserung)) 2 study [45], after three months of treatment, a 25% mean decrease in NRS was reported in 75 patients. The MOVE 2 EU multicentric study utilized the same protocol, but patients were recruited from Italy, Norway and Denmark [46]. Similarly, the mean decrease in spasticity NRS was 22% in 265 patients. The spasticity NRS decrease was available in only 22% and 61% of the cases, respectively.

The effectivity of nabiximols is further underlined by the significant improvement in the spasticity-related parameters, the results of the 10-m walking test, and sleep disturbance. To a lesser extent, mAS values are also in accordance with the primary results, but similarly to a previous meta-analysis [7], a significant improvement could not be detected.

However, in terms of the Barthel ADL score, patients treated with nabiximols showed no significant changes or in some cases even worsening. This can be explained in a broader sense by the pathomechanism of spasticity and the aspecific nature of this test method. The Barthel ADL assesses the feasibility of daily routine activities, and the functional independence of the patient and is therefore a non-specific measure of spasticity. Since the performance of the activities requires dynamic interaction of basic neuronal functional systems (vestibular, somatosensory, motor and autonomic), the Barthel Index cannot be adapted exclusively to the assessment of spasticity. The development of spasticity is due to a primary central nervous system motor deficit, *i.e*. motor dysfunction in patients with spasticity greatly modifies the overall Barthel Index score [47]. Another particular point in the Barthel ADL is while it measures aspects of functional independence, the index calculation may differ across the studies (Table **S1f**). The results of Novotna *et al*. (2011) increased the risk of bias for Barthel ADL since they were highly different from the others and no clear definition of the scaling was provided [37].

While Barthel ADL may rise attention to side effects, significant improvement in the SGIC highlights the overall beneficial effect of nabiximols which were also reported as generally well tolerated by the patients in all the identified studies.

Data about quality of life were scarce and very heterogeneous. Most of the studies reported little impact of nabiximols on quality of life parameters, probably because of the other MS symptoms that were not ameliorated by nabiximols as emphasized by Markova *et al*. (2019) [39].

Overall, the favorable effects of nabiximols on spasticity and, in some cases, spasticity-related measures are proven, implying that improvements are statistically and clinically significant.

### 4.1 Strength and Limitations

The most prominent strength of our study is that the results are based on randomized, double-blinded studies hence summarizing the highest level of evidence. Blinding is especially important in patient-reported parameters like NRS, because patient’s beliefs can distort the results. Another advantage of our meta-analysis is the robust methodology. It should be also mentioned that this study is the most recent meta-analysis in the topic.

On the other hand, there are several limitations. While for some of the outcomes the estimated heterogeneity was low, the results should be interpreted cautiously, because of the low number of available studies.

The different study designs contribute to the heterogeneity since enriched study design and cross-over study protocols have an impact on the outcomes. The treatment duration also markedly differed spanning from 3 to 14 weeks. On the other hand, this may have only a low impact on our findings, because the most prominent changes in MS-related spasticity and pain are present in the first 3-4 weeks [6, 36, 37, 39, 40]. Moreover, similar effects could be observed in the analysis of short-term and long-term studies by us as well.

Another limitation was the need to re-calculate the results for some of the reported outcomes to be able to perform a pooled analysis. We would like to highlight the risk of bias analysis results as well. All data were from articles that posed at least some concerns. Moreover, all studies were sponsored by the producer/distributor of Sativex^®^.

### 4.2 Implications for Research and Clinical Practice

Despite the numerous limitations of the study, nabiximols have a clear effect on resistant MS spasticity, therefore, it can be a relevant treatment option in this clinical scenario.

However, it is still a question, of how it should be implemented, and what is the best target population. In this regard, Caroteuto and coworkers (2020) already noticed [48] that nabiximols may have a better effect if the treatment is started early during the disease course. Furthermore, the effect of the drug may also differ depending on the phenotype of MS.

Lastly, well-designed comparisons with other antispastic agents are scarce, especially with enriched study design, therefore the sequence of antispastic therapies needs investigation.

## CONCLUSION

Our meta-analysis underlines that nabiximols alleviate MS-associated spasticity when administered as an add-on in patients not responding well to standard treatment. On the other hand, the side effects and limitations of our study (*e.g*. different study designs, high heterogeneity, risks of bias) should not be underestimated.

Future studies will be necessary to define the optimal population.

## Figures and Tables

**Fig. (1):**
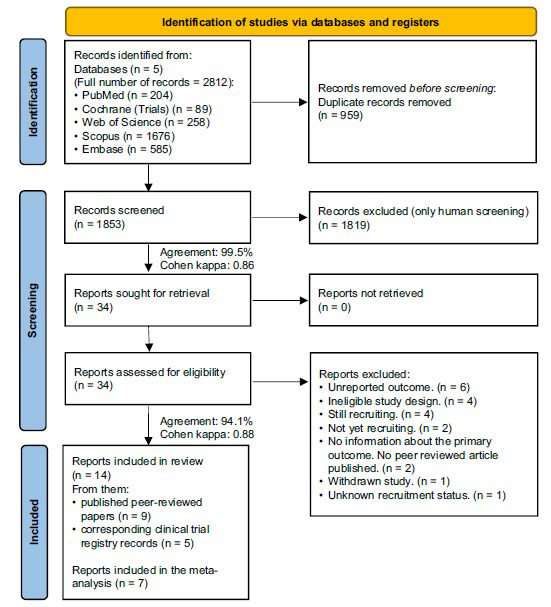
Flow diagram of study identification and selection by PRISMA 2020.

**Fig. (2):**
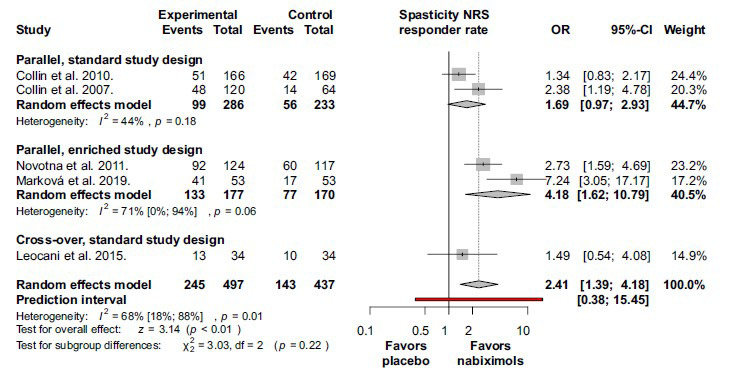
Nabiximols alleviates multiple sclerosis associated spasticity more effectively than placebo in terms of responder rates. Treatment responders (events) are the number of patients with clinically relevant alleviation in spasticity, defined in spasticity numerical rating scale. Response was defined as a 30% reduction in NRS at measurement moment in all studies except Leocani *et al*. that considered a 20% decrease of NRS as response. **Abbreviations**: Confidence interval (CI); numerical rating scale (NRS); odds ratio (OR).

**Table 1:** Baseline characteristics of studies included in the review: Trial characteristics.

**Author**	**Centers (Country)**	**Study Design**	**Length of DB Phase (Weeks)**	**Outcomes**
Wade *et al.* 2004 [34]	3 (UK)	R, DB, PD, PC	6	Spasticity VAS, TW; mAS; sleep disturbance VAS; Barthel ADL; SGIC
Collin *et al*. 2007 [6]	8 (UK); 4 (RO)	R, DB, PD; PC	6	Spasticity responder rate; spasticity NRS; AS; SGIC
Aragona *et al*. 2009 [35]	1 (IT)	R, DB, PC, CO	2x3	TW; VAS QoL
Conte *et al*. 2009 [41]	1 (IT)	R, DB, PC, CO	2x3	Spasticity NRS; AS
Collin *et al*. 2010 [36]	15 (UK); 8 (CR)	R, DB, PD; PC	14	Spasticity responder rate; spasticity NRS; TW; mAS; sleep disturbance NRS; Barthel ADL; EQ-5D & MSQoL-54
Novotna *et al*. 2011 [37]	18 (UK); 11 (SP); 10 (PO); 8 (CR); 5 (IT)	R; ED; PC	12	Spasticity responder rate; spasticity NRS; TW; mAS; sleep disturbance NRS; Barthel ADL; SGIC; EQ-5D; SF-36
Langford *et al*. 2013 [40]	12 (UK); 7 (CR); 5 (CA); 5 (SP); 4 (FR)	R; DB; PD; PC	14	Spasticity NRS; sleep disturbance NRS; EQ-5D; SF-36
Leocani *et al*. 2015 [38]	1 (IT)	R; DB; PC; CO	2x4	Spasticity responder rate*; spasticity NRS; TW; mAS; sleep disturbance NRS
Marková *et al*. 2019 [39]	14 (CR); 1 (AU)	R; ED; PC	12	Spasticity responder rate; spasticity NRS; TW; mAS; sleep disturbance NRS; Barthel ADL; SGIC; SF-36

**Note:** Data provided here are the characteristics of double-blind phases. From trials with enriched study design we presented data from DB phases, exceptions are marked (c).

Countries: Austria (AU); Canada (CA); Czech Republic (CR); France (FR); Italy (IT); Poland (PO); Romania (RO); Spain (SP); United Kingdom (UK). Study design: cross-over (CO); double-blind (DB); enriched design (consists of an SB and a DB phase) (ED); randomized (R), placebo-controlled (PC); parallel design (PD); single-blind (SB).

Outcomes: Activities of Daily Living (ADL); Ashworth scale (AS); modified Ashworth scale (mAS); Multiple Sclerosis Quality of Life-54 (MSQoL-54); quality of life (QoL); Subject's Global Impression of Change (SGIC); timed walking test (regardless of distance) (TW); visual analog scale (VAS); 36-Item Short Form Survey (SF-36).

Other: multiple sclerosis (MS); standard deviations (SD); standard error (SE). **Note**: *altered cut-off point (20% instead of 30%).

**Table 2:** Baseline characteristics of studies included in the review: Patient characteristics.

**Author**	**Medication**	**Number of Patients**	**Sex (Female % of Total)**	**Age (Years;** **Mean ± SD)**	**Duration of MS (Years; Mean ± SD)**	**Baseline Spasticity NRS (Mean ± SD)**	**General Daily Actuation ** **(Mean ± SD)**	**Disease ** **Phenotype (%)**
Wade *et al.* 2004 [34]	Sativex^®^	80	58.8	51.0 ± 9.4	no data	no data	14.6 ± 1.4^d^	no data
	Placebo	80	65.0	50.4 ± 9.3	no data	no data	23.4 ± 1.8^d^	
Collin *et al*. 2007 [6]	Sativex^®^	124	60.3	49.7 ± 10.2	13.6 ± 8.6	5.49	9.4 ± 6.4	no data
	Placebo	65	52.3	47.8 ± 9.5	12.2 ± 7.7	5.39	14.7 ± 8.4	
Aragona *et al*. 2009 [35]	Sativex^®^	17^b^	64.7	49.8 ± 6.6	20.8 ± 8.4	no data	15.2 ± 4.5	100% SPMS
	Placebo	17^b^	64.7	49.8 ± 6.6	20.8 ± 8.4	no data	8.2 ± 3.15	
Conte *et al*. 2009 [41]	Sativex^®^	18^b^	66.7	51.1 ± 5.3	22.2 ± 8.9	no data	8.0^e^ (2.7-12.5)^f^	100% SPMS
	Placebo	18^b^	66.7	51.1 ± 5.3	22.2 ± 8.9	no data	15.7^e^ (6.7-26)^f^	
Collin *et al*. 2010 [36]	Sativex^®^	167	63.5	48.0 ± 10.6	14.4 ± 8.3	6.77 ± 0.10^d^	8.5 (1-22)^f^	no data
	Placebo	170	59.4	47.1 ± 9.2	16.0 ± 8.5	6.48 ± 0.10^d^	15.4 (2-23)^f^	
Novotna *et al*. 2011 [37]	Sativex^®^	124	58.1	49.1 ± 9.1	13.3 ± 8.3	6.91 ± 1.25^c^	6.9 ± 1.8^c^ 8.3 ± 2.4	no data
	Placebo	117	62.4	48.1 ± 9.6	11.8 ± 7.4		8.9 ± 2.4	
Langford *et al*. 2013 [40]	Sativex^®^	167	67.7	48.4 ± 10.4	11.4 ± 8.0	not defined	8.8 ± 3.9	12% PPMS; 40% SPMS; 46% RRMS; 2% PRMS
	Placebo	172	68.0	49.5 ± 10.5	12.5 ± 8.5	not defined	11.1 ± 4.6	
Leocani *et al*. 2015 [38]	Sativex^®^	34^b^	44.1	48 ± 7	17.3 ± 8.4	7.1 ± 1.4	7 ± 3	100% PPMS or SPMS
	Placebo^a^	34^b^	44.1	48 ± 7	17.3 ± 8.4	7.1 ± 1.4	10 ± 3	
Marková *et al*. 2019 [39]	Sativex^®^	191^c^; 53	70.2^c^	51.3 ± 10.2^c^	14.2 ± 8.4^c^	6.4 ± 1.2^c^	7.7 ± 3.0^c^; 7.3 ± 2.7	11% PPMS^c^; 48% SPMS^c^; 41% RRMS^c^
	Placebo	53					8.5 ± 3.0	

**Note:** Data provided here are the characteristics of double-blind phases. From trials with enriched study design we presented data from DB phases, exceptions are marked (c).

The phenotype of MS: primary progressive MS (PPMS); progressive relapsing MS (PRMS); relapsing-remitting MS (RRMS); secondary progressive MS (SPMS).

Other: multiple sclerosis (MS); standard deviations (SD); standard error (SE). a: does not clarify whether it was identical; b: cross-over design; c: data from enriched design study's SB phase; d: SE was given instead of SD; e: median; f: range.

## LIST OF ABBREVIATIONS

ADL: Activities of Daily Living
AS: Ashworth Scale
CI: Confidence Interval
mAS: Modified Ashworth Scale
MD: Mean Difference
MS: Multiple Sclerosis
NRS: Numerical Rating Scale
OR: Odds Ratio
SF-36: 36-Item Short Form Survey
SGIC: Subject Global Impression of Change
VAS: Visual Analog Scale

## SUPPLEMENTARY MATERIAL

PRISMA checklist is available as supplementary material on the publisher’s website along with the published article.
