# Supplementary material for: Nabiximols is Efficient as Add-On Treatment for Patients with Multiple Sclerosis Spasticity Refractory to Standard Treatment: A Systematic Review and Meta-Analysis of Randomised Clinical Trials
Source: Curr Neuropharmacol. 2023 Sep 25;21(12):2505–15. doi: 10.2174/1570159X21666230727094431 (PMC10616923; doi:10.2174/1570159X21666230727094431)
Supplement: Supplementary file 1 [file CN-21-2505_SD1.pdf]

Supplementary Material

Nabiximols is Efficient as Add-On Treatment for Patients with Multiple Sclerosis Spasticity Refractory to Standard Treatment: A Systematic Review and Meta-Analysis of Randomised Clinical Trials

Dénes Kleiner[1,2], István László Horváth[1,2], Stefania Bunduc[2,3,4,5], Dorottya Gergő[2,6], Katalin Lugosi[2,7], Péter Fehérvári[2,8], Péter Hegyi[2,3,9,10] and Dezső Csupor[2,10,11,12,*]

[1]University Pharmacy, Department of Pharmacy Administration, Semmelweis University, Hőgyes Endre utca 7-9., 1092 Budapest, Hungary; [2]Centre for Translational Medicine, Semmelweis University, Üllői út 26, 1085, Budapest, Hungary; [3]Division of Pancreatic Diseases, Heart and Vascular Center, Semmelweis University, Baross út 22-24, 1085 Budapest, Hungary; [4]Faculty of Medicine, Carol Davila University of Medicine and Pharmacy, Dionisie Lupu Street 37, 020021, Bucharest, Romania; [5]Center of Digestive Disease and Liver Transplant, Fundeni Clinical Institute, Fundeni Street 258, 022328, Bucharest, Romania; [6]Department of Pharmacognosy, Semmelweis University, Üllői út 26., 1085 Budapest, Hungary; [7]Department of Neurology, Bajcsy-Zsilinszky Hospital, Maglódi Road 89-91, 1106 Budapest, Hungary; [8]Budapest Department of Biostatistics, University of Veterinary Medicine, István utca 2., 1078 Budapest, Hungary; [9]János Szentágothai Research Center, University of Pécs, Szigeti út 12, 7624 Pécs, Hungary; [10]Institute for Translational Medicine, Medical School, University of Pécs, Szigeti út 12, 7624 Pécs, Hungary; [11]Institute of Clinical Pharmacy, University of Szeged, Szikra utca 8, 6725 Szeged, Hungary; [12]Department of Pharmacognosy, University of Szeged, Eötvös u. 6, 6720 Szeged, Hungary

1875-6190/23 $65.00+.00 **© 2023 Bentham Science Publishers**

Table S1a. The evaluation of Cohen's kappa coefficient (k)

k	Agreement level
≤ 0	no agreement
0.10-0.20	none or slight
0.21-0.40	fair agreement
0.41-0.60	moderate agreement
0.61-0.80	substantial agreement
0.81-1.00	almost perfect

Table S1b. For the evaluation of spasticty NRS recalculations had to be performed for a study

Author	Measurement of spasticity	Minimum	Maximum	Recalculation for the statistical analysis
Wade et al 2004.	VAS	0	100	[published VAS]/10
Collin et al. 2007.	NRS	0	10	no recalculation
Collin et al. 2010.	NRS	0	10	no recalculation
Novotna et al. 2011.	NRS	0	10	no recalculation
Marková et al. 2019.	NRS	0	10	no recalculation

Abbreviations: NRS: numerical rating scale; VAS: visual analog scale.

Table S1c. For the evaluation of 10-m walking test recalculations had to be performed for a study

**Article**	**Distance**	**Unit of Measure**	**Recalculation for the statistical analysis**
Wade et al 2004.	10 m	sec	no recalculation
Aragona et al. 2009	25 ft	sec	10*[published distance]/7.62
Collin et al. 2010.	10 m	sec	no recalculation
Leocani et al. 2015.	10 m	sec	no recalculation
Marková et al. 2019.	10 m	sec	no recalculation
Novotná et al. 2011.	10 m	sec	no recalculation

Table S1d. For the evaluation of mAS recalculations had to be performed for a study.

Article	Number of assessed muscle groups	Scale's minimum at a muscle group	Scale's maximum at a muscle group	Minimum of total score	Maximum of total score	Recalculation for the statistical analysis
Wade et al 2004	20	1	5	20	100	no recalculation
Collin et al. 2010.	20	1	5	20	100	no recalculation
Novotna et al. 2011.	20	1	5	20	100	no recalculation
Marková et al. 2019.	10	0	5	0	50	80*[published mAS]/50

Abbreviation: mAS: modified Ashworth scale

Table S1e. For the evaluation of spasticty NRS recalculations had to be performed for a study.

Author	Measurement of spasticity	Minimum	Maximum	Recalculation for the statistical analysis
Wade et al. 2004.	VAS	0	100	[published VAS]/10
Collin et al. 2010.	NRS	0	10	no recalculation
Novotna et al. 2011.	NRS	0	10	no recalculation
Leocani et al. 2015.	NRS	0	10	no recalculation
Marková et al. 2019.	NRS	0	10	no recalculation

Abbreviations: NRS: numerical rating scale; VAS: visual analog scale

Table S1f. For the evaluation of Bartel ADL recalculations had to be performed for two studies.

Author	Number of items	Minimum of an item	Maximum of an item	Minimum of total score	Maximum of total score	Recalculation for the statistical analysis
Wade et al 2007.	10	0	1 or 2 or 3	0	20	no recalculation
Collin et al. 2010.	10	no data	no data	no data	100	20*[published Barthel ADL score]/100
Novotna et al. 2011.	no data	no data	no data	no data	no data	20*[published Barthel ADL score]/100
Marková et al. 2019.	10	0	1 or 2 or 3	0	20	no recalculation

Abbreviations: ADL: activities of daily living

Table S1g. Evaluation of SGIC

State	Count as
very much improved (better)	responder
much improved (better)	
slightly improved (better)	non responder
no change	
slightly worse	
much worse	
very much worse	

Abbreviations: SGIC: subject's global impression of change

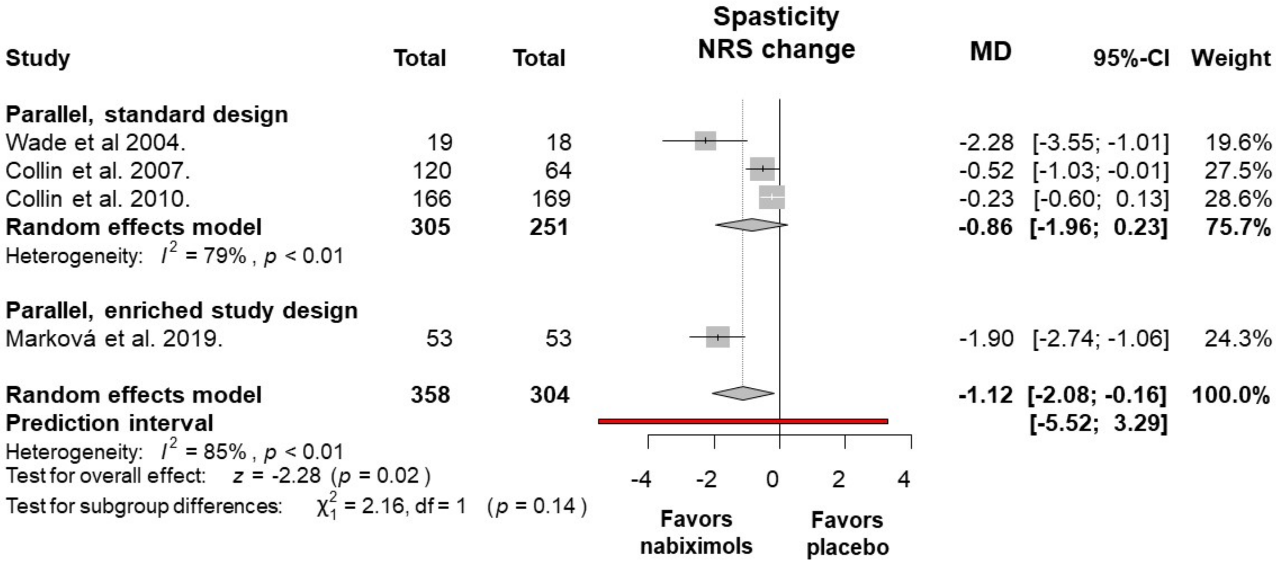

Fig. (S1). Nabiximols alleviates multiple sclerosis associated spasticity more effectively than placebo in terms of symptom severity measured by numerical rating scale in long-term studies (treatment duration ≥ 6 weeks).
For this analysis Novotna et al. (2011) was excluded, because of the differing baseline.
Abbreviations: CI: confidence interval; MD: mean difference; NRS: numerical rating scale

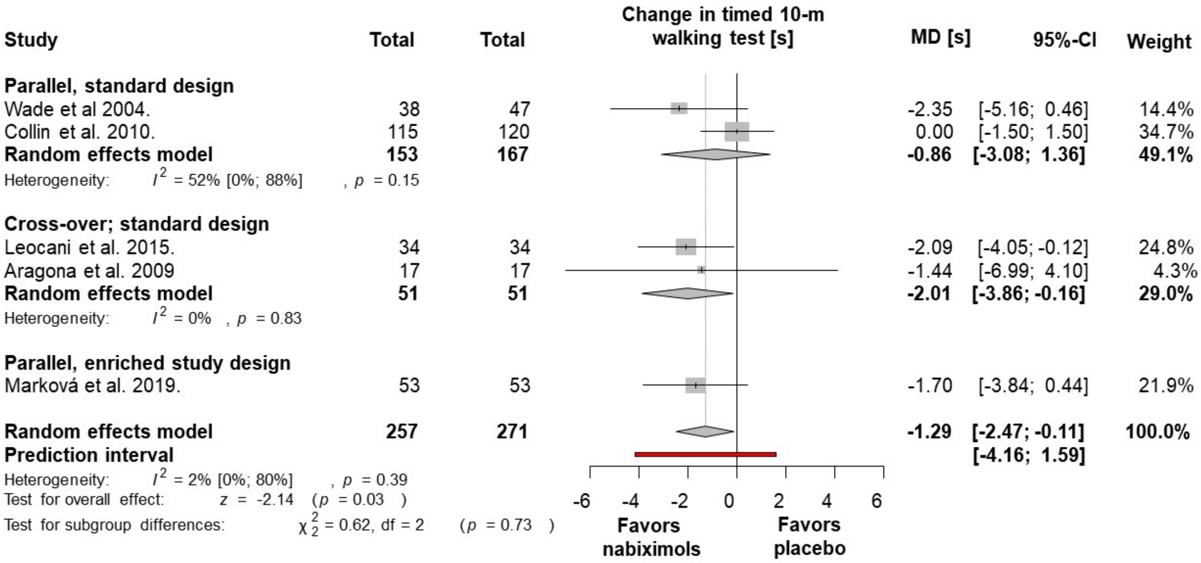

Fig. (S2). Nabiximols improves gait, measured by 10-m timed walking test.
For this analysis Novotna et al. (2011) was excluded, because of the differing baseline.
Abbreviations: CI: confidence interval; MD: mean difference

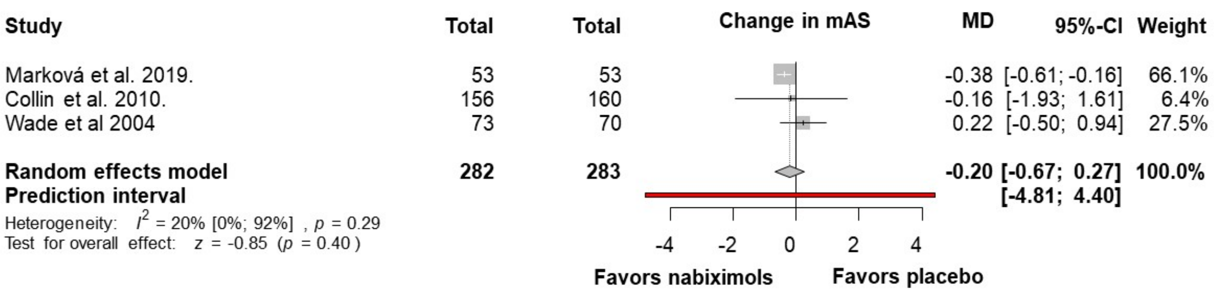

Fig. (S3.) Nabiximols improves non-significantly spasticity, measured by mAS.
For this analysis Novotna et al. (2011) was excluded, because of the differing baseline.
Abbreviations: CI: confidence interval; mAS: modified Ashworth scale; MD: mean difference

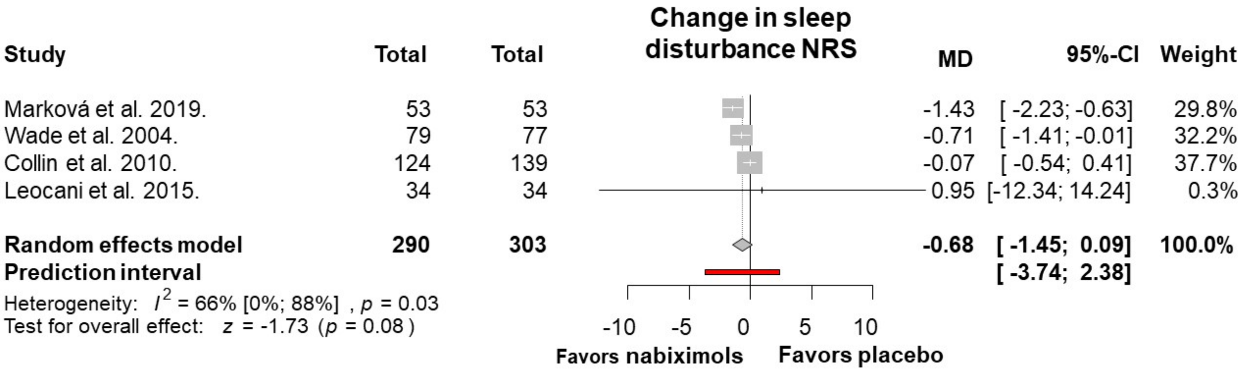

Fig. (S4.) Sleep disturbance was ameliorated by nabiximols in patients treated with nabiximols by comparison with placebo.
For this analysis Novotna et al. (2011) was excluded, because of the differing baseline.
Abbreviations: CI: confidence interval; MD: mean difference; NRS: numerical rating scale

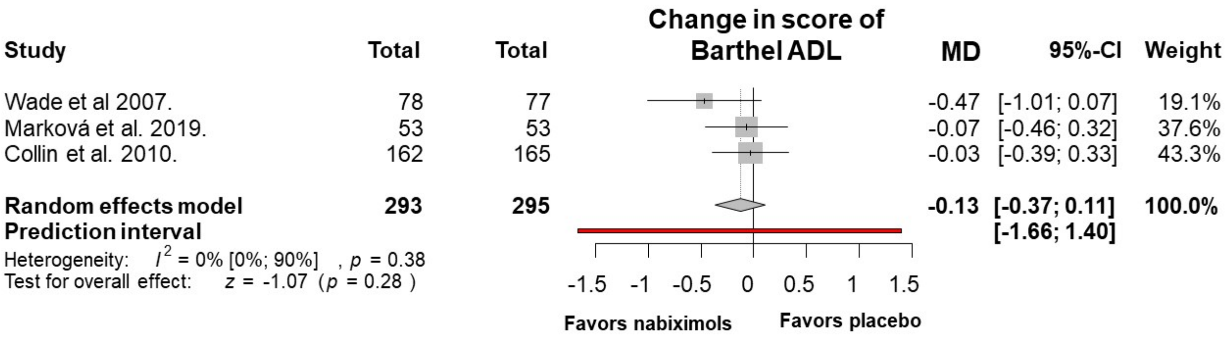

Fig. (S5). The Barthel activities of daily living does not change significantly.
For this analysis Novotna et al. (2011) was excluded, because of the differing baseline.
Abbreviations: ADL: activities of daily living; CI: confidence interval; MD: mean difference

Study ID	Weight	Randomisation process	Deviations from the intended interventions	Missing outcome data	Measurement of the outcome	Selection of the reported result	Overall
Collin et al. 2007	1	!	+	+	+	+	!
Collin et al. 2010	1	!	+	+	+	+	!
Novotna et al. 2011	1	!	+	+	+	+	!
Leocani et al. 2015	1	!	+	+	+	+	!
Markovà et al. 2019	1	!	+	+	+	+	!

+ Low risk
! Some concerns
- High risk

Fig. (S6a.) Risk of bias assessment of spasticity NRS responder rate. The revised Cochrane risk-of-bias tool (RoB2) was used.
Abbreviation: NRS: Numerical rating scale

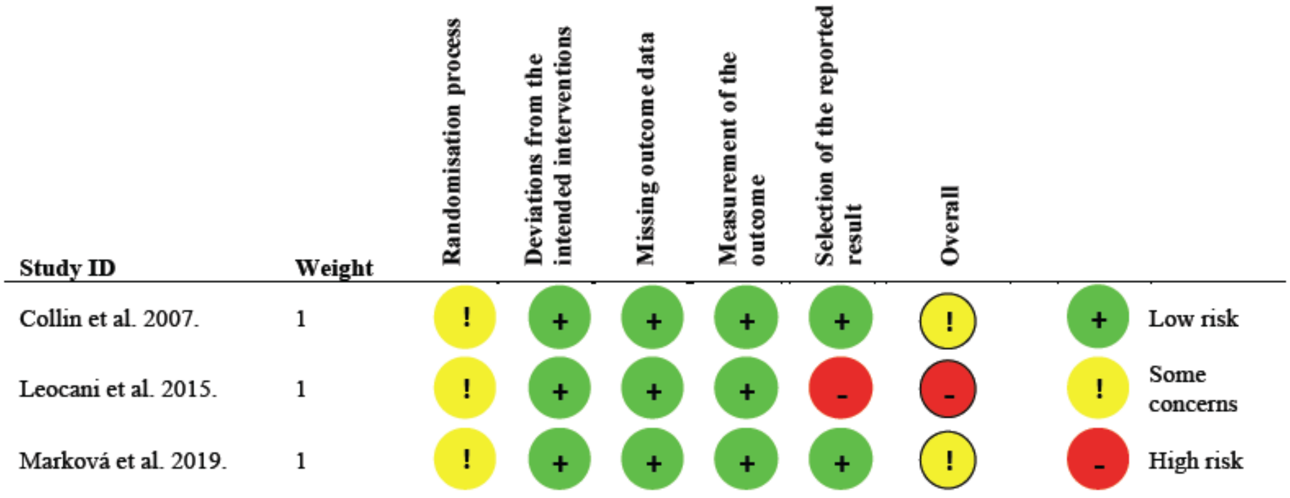

Fig. (S6b). Risk of bias assessment of spasticity NRS short-term decrease. The revised Cochrane risk-of-bias tool (RoB2) was used.
Abbreviation: NRS: Numerical rating scale

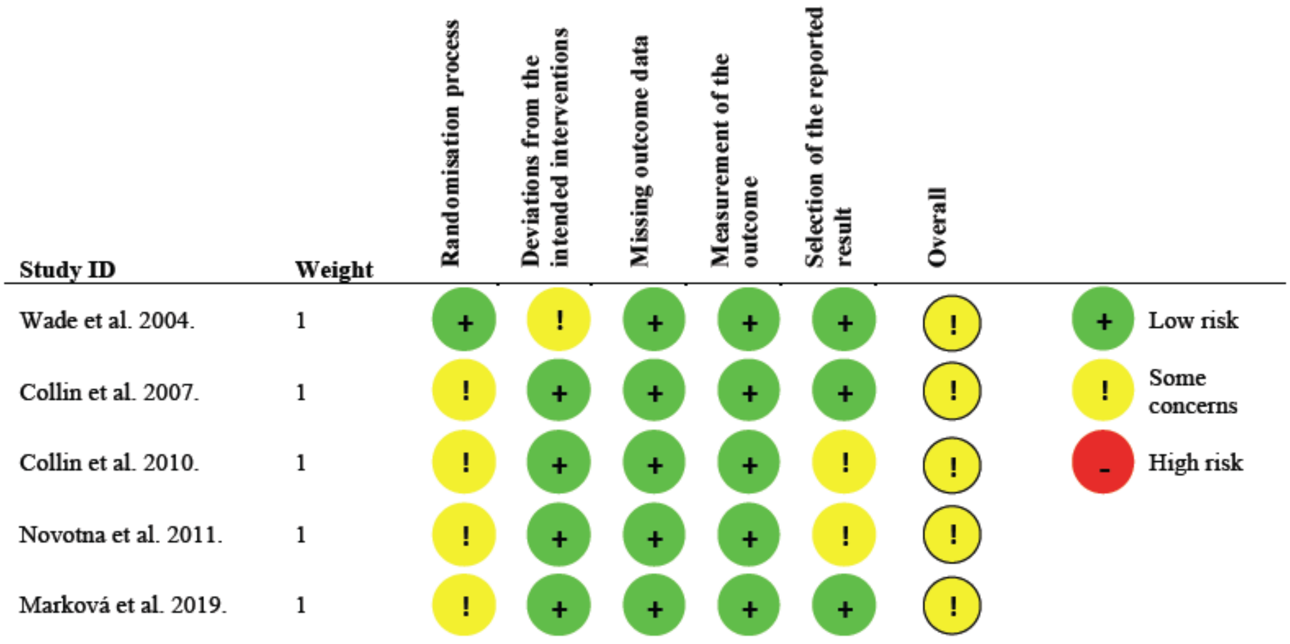

Fig. (S6c). Risk of bias assessment of long-term decrease in spasticity NRS. The revised Cochrane risk-of-bias tool (RoB2) was used.
Abbreviation: NRS: Numerical rating scale

Study ID	Weight	Randomisation process	Deviations from the intended interventions	Missing outcome data	Measurement of the outcome	Selection of the reported result	Overall
Wade et al. 2004.	1	+	!	-	+	+	-
Aragona et al. 2009.	1	!	+	+	+	!	!
Collin et al. 2010.	1	!	+	-	+	-	-
Novotna et al. 2011.	1	!	+	-	+	-	-
Leocani et al. 2015.	1	!	+	+	+	-	-
Markovà et al. 2019.	1	!	+	+	+	+	!

+ Low risk
! Some concerns
- High risk

Fig. (S6d). Risk of bias assessment of timed walking test. The revised Cochrane risk-of-bias tool (RoB2) was used.

Study ID	Weight	Randomisation process	Deviations from the intended interventions	Missing outcome data	Measurement of the outcome	Selection of the reported result	Overall
Wade et al. 2004.	1	+	-	!	+	!	-
Collin et al. 2010.	1	!	+	-	+	-	-
Novotna et al. 2011.	1	!	+	+	+	!	!
Markovà et al. 2019.	1	!	+	+	+	+	!

+ Low risk
! Some concerns
- High risk

Fig. (S6e). Risk of bias assessment of mAS. The revised Cochrane risk-of-bias tool (RoB2) was used.
Abbreviations: mAS: modified Ashworth scale

Study ID	Weight	Randomisation process	Deviations from the intended interventions	Missing outcome data	Measurement of the outcome	Selection of the reported result	Overall
Wade et al. 2004.	1	+	!	+	+	-	-
Collin et al. 2010.	1	!	+	-	+	!	-
Novotna et al. 2011.	1	!	+	+	+	-	-
Leocani et al. 2015.	1	!	+	+	+	-	-
Markovà et al. 2019.	1	!	+	+	+	+	!

+ Low risk
! Some concerns
- High risk

Fig. (S6f). Risk of bias assessment of sleep disruption. The revised Cochrane risk-of-bias tool (RoB2) was used.

Study ID	Weight	Randomisation process	Deviations from the intended interventions	Missing outcome data	Measurement of the outcome	Selection of the reported result	Overall
Wade et al. 2004.	1	+	!	+	+	!	!
Collin et al. 2010.	1	!	+	+	+	-	-
Novotna et al. 2011.	1	!	-	-	+	-	-
Markovà et al. 2019.	1	!	+	+	+	+	!

+ Low risk
! Some concerns
- High risk

Fig. (S6g). Risk of bias assessment of Barthel ADL. The revised Cochrane risk-of-bias tool (RoB2) was used.
Abbreviations: ADL: activities of daily living

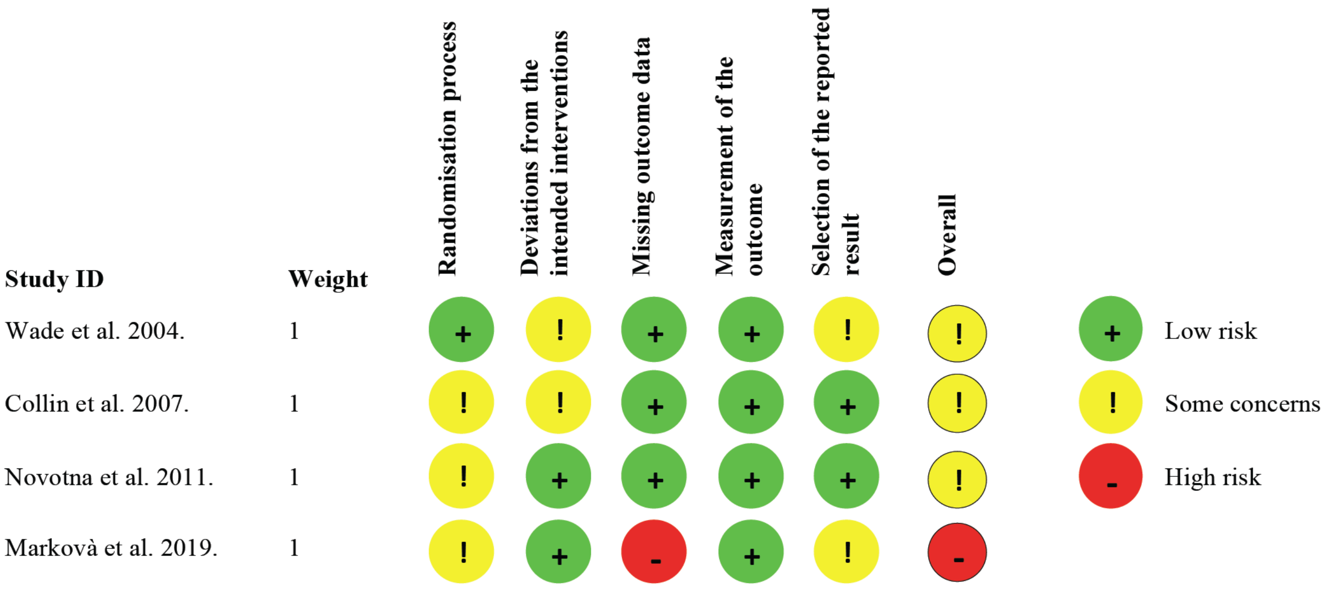

Fig. (S6h). Risk of bias assessment of SGIC. The revised Cochrane risk-of-bias tool (RoB2) was used.
Abbreviations: SGIC: subject's global impression of change

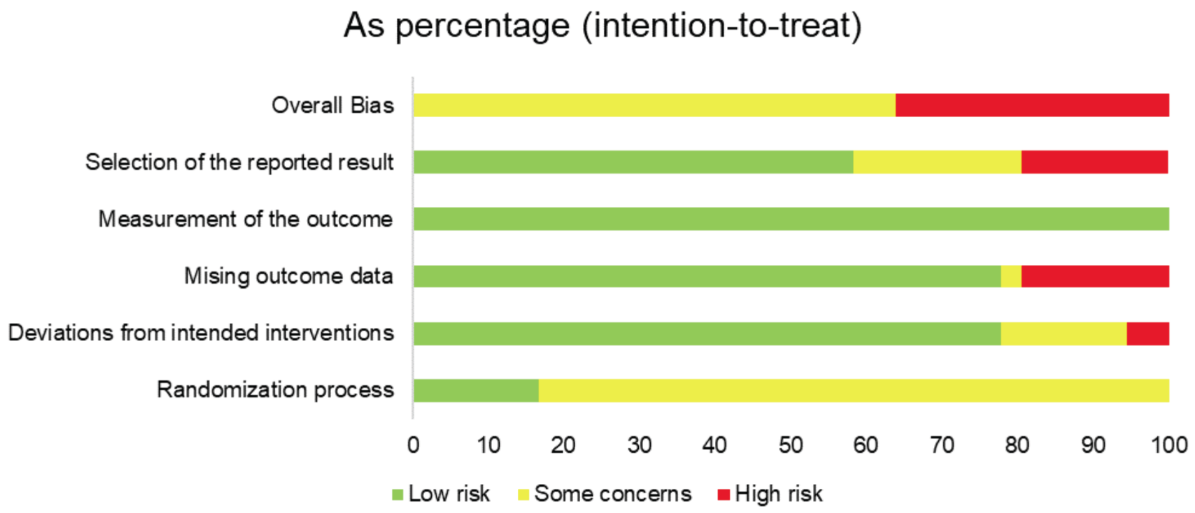

Fig. (S7). Summary of the risk of bias assessment.
The revised Cochrane risk-of-bias tool (RoB2) was used.

Table S2. Quality of life parameters reported across the eligible studies.

Study	Tool	Effect size nabiximols (mean ± SD)	Effect size placebo (mean ± SD)	*p*
Aragona et al. 2009.[a]	VAS QoL – before treatment	4.29 ± 2.08	4.29 ± 2.08	
	VAS QoL – after treatment	4.00 ± 2.00	3.65 ± 2.29	0.31
Collin et al. 2010.	EQ-5D; health state index	0.03	0.01	0.175
	EQ-5D; health status VAS	4.29	2.87	0.538
	MSQoL-54; physical health	5.10	6.61	0.549
	MSQoL-54; mental health	-0.05	3.04	0.312
Novotna et al. 2011.	EQ-5D; health state index	0.003 ± 0.155 (article: -0.03)[c]	-0.013 ± 0.176 (article: -0.05)[c]	0.284
	EQ-5D; health status VAS	-0.7 ± 15.3 (article: -1.99)	-2.8 ± 19.9 (article: -3.24)	0.564
	SF-36; physical functioning	0.30	0.76	0.782
	SF-36; role physical	-0.31	0.98	0.658
	SF-36; bodily pain	-0.05	-5.06	0.060
	SF-36; general health	1.20	-0.12	0.442
	SF-36; vitality	-1.17	-3.35	0.306
	SF-36; social functioning	-0.97	-0.32	0.840
	SF-36; role emotion	-1.26	1.53	0.343
	SF-36; mental health	-2.20	-2.94	0.683
Langford et al. 2013.	EQ-5D; health state index	0.05	0.07	0.396
	EQ-5D; health status VAS	7.20	5.26	0.383
	SF-36; physical functioning	1.56	2.02	0.785
	SF-36; role physical	5.62	6.51	0.694
	SF-36; bodily pain	11.36	10.01	0.494
	SF-36; general health	2.32	4.02	0.264
	SF-36; vitality	3.72	6.47	0.095
	SF-36; social functioning	3.62	9.37	0.020
	SF-36; role emotion	-0.18	3.15	0.216
	SF-36; mental health	3.17	3.73	0.733
Marková et al. 2019.	SF-36; physical functioning[b]	4.08 [95%CI 0.57 - 7.59]	3.65 [95%CI (-0.05)-7.35]	0.868
	SF-36; role physical[b]	7.44 [95%CI (2.99) - 11.89)	4.77 [95%CI 0.08-9.46]	0.415
	SF-36; bodily pain[b]	19.71 [95%CI 14.34 - 25.09)	10.41 [95%CI 4.74-16.08]	0.020
	SF-36; general health[b]	0.31 [95%CI -3.71 - 4.34)	1.90 [95%CI (-2.34)-6.15]	0.591
	SF-36; vitality[b]	8.34 [95%CI 3.89 - 12.78)	3.00 [95%CI (-1.69)-7.68]	0.104
	SF-36; social functioning[b]	7.68 [95%CI 3.12 - 12.24]	4.27 [95%CI (-0.54)-9.08]	0.311
	SF-36; role emotion[b]	6.21 [95%CI 1.32 - 11.11]	4.99 [95%CI (-0.18)-10.16]	0.734
	SF-36; mental health[b]	5.52 [95%CI 2.09 - 8.95]	3.38 [95%CI (-0.23)-7.00]	0.398

Abbreviations: CI: confidence interval; SD: standard deviation; SF-36: 36-Item short form health survey; VAS: visual analog scale; VAS QoL: visual analog scale for quality of life; MSQoL-54: Multiple Sclerosis Quality of Life-54

[a]: only before-after data was provided; [b]: instead of SD CI was published; [c]: because of the prominent difference between the registry and the article's result, we have provided all available results, and highlighted those, that are from the article

Table S3. Evaluation of evidence level by Grading of Recommendations, Assessment, Development and Evaluations (GRADE) framework.

Certainty assessment							№ of patients		Effect		Certainty	Importance
№ of studies	Study design	Risk of bias	Inconsistency	Indirectness	Imprecision	Other considerations	nabiximols	placebo	Relative (95% CI)	Absolute (95% CI)		
**Treatment responders in spasticity NRS**												
5	randomized trials	not serious	serious[a,b]	not serious	not serious	none	245/497 (49.3%)	143/437 (32.7%)	**OR 2.41** (1.39 to 4.18)	**21 more per 100** (from 8 more to 34 more)	⊕⊕⊕◯ Moderate	CRITICAL
**Decrease in spasticity NRS (short-term treatment)**												
3	randomized trials	serious	serious[a]	not serious	not serious	none	207	151	-	**0** (0 to 0)	⊕⊕◯◯ Low	IMPORTANT
**Decrease in spasticity NRS (long-term treatment) (follow-up: range 6 weeks to 16 weeks)**												
5	randomized trials	not serious	serious[a,c]	not serious	not serious	none	482	421	-	**0** (0 to 0)	⊕⊕⊕◯ Moderate	CRITICAL
**Timed walk**												
6	randomized trials	very serious	very serious[a]	serious	not serious	none	341	354	-	**0** (0 to 0)	⊕◯◯◯ Very low	IMPORTANT
**mAS**												
4	randomised trials	very serious	very serious[a,d]	serious	not serious	none	403	399	-	**0** (0 to 0)	⊕◯◯◯ Very low	NOT IMPORTANT
**Sleep disturbance NRS**												
5	randomised trials	very serious	serious[a,e]	serious	not serious	none	414	420	-	**0** (0 to 0)	⊕◯◯◯ Very low	NOT IMPORTANT
**Barthel ADL**												
4	randomised trials	very serious	serious[a,f]	serious	not serious	none	417	412	-	**0** (0 to 0)	⊕◯◯◯ Very low	IMPORTANT
**SGIC responder rate**												
4	randomised trials	serious	serious[a,d]	serious	not serious	none	120/367 (32.7%)	72/304 (23.7%)	**OR 1.72** (1.21 to 2.46)	**11 more per 100** (from 4 more to 20 more)	⊕◯◯◯ Very low	NOT IMPORTANT

Explanations of down-grading: a. Low number of retracted studies; b. Relatively high heterogenity [I^2=68% (p=0.01)].; c. Relatively high heterogenity [I^2 = 81% ($p < 0.01$)]; d. Enormous differences between weight of studies underpins inconsistency between studies.; e. Relatively high heterogeneity [I^2 = 63% (p = 0.03)]; f. Relatively high heterogeneity [I^2 = 76% ($p < 0.01$)].

Abbreviations: ADL: activities of daily living; CI: confidence interval; mAS: modified Ashworth scale; NRS: Numerical rating scale; OR: odds ratio; SGIC: subject's global impression of change

PRISMA 2020 Checklist

Section and Topic	Item #	Checklist Item	Location where Item is Reported
**TITLE**			
Title	1	Identify the report as a systematic review.	1
**ABSTRACT**			
Abstract	2	See the PRISMA 2020 for Abstracts checklist.	1
**INTRODUCTION**			
Rationale	3	Describe the rationale for the review in the context of existing knowledge.	1-2
Objectives	4	Provide an explicit statement of the objective(s) or question(s) the review addresses.	2
**METHODS**			
Eligibility criteria	5	Specify the inclusion and exclusion criteria for the review and how studies were grouped for the syntheses.	2
Information sources	6	Specify all databases, registers, websites, organisations, reference lists and other sources searched or consulted to identify studies. Specify the date when each source was last searched or consulted.	2
Search strategy	7	Present the full search strategies for all databases, registers and websites, including any filters and limits used.	2
Selection process	8	Specify the methods used to decide whether a study met the inclusion criteria of the review, including how many reviewers screened each record and each report retrieved, whether they worked independently, and if applicable, details of automation tools used in the process.	2
Data collection process	9	Specify the methods used to collect data from reports, including how many reviewers collected data from each report, whether they worked independently, any processes for obtaining or confirming data from study investigators, and if applicable, details of automation tools used in the process.	2
Data items	10a	List and define all outcomes for which data were sought. Specify whether all results that were compatible with each outcome domain in each study were sought (e.g. for all measures, time points, analyses), and if not, the methods used to decide which results to collect.	2-3
	10b	List and define all other variables for which data were sought (e.g. participant and intervention characteristics, funding sources). Describe any assumptions made about any missing or unclear information.	2-3; 10
Study risk of bias assessment	11	Specify the methods used to assess risk of bias in the included studies, including details of the tool(s) used, how many reviewers assessed each study and whether they worked independently, and if applicable, details of automation tools used in the process.	3
Effect measures	12	Specify for each outcome the effect measure(s) (e.g. risk ratio, mean difference) used in the synthesis or presentation of results.	2-3
Synthesis methods	13a	Describe the processes used to decide which studies were eligible for each synthesis (e.g. tabulating the study intervention characteristics and comparing against the planned groups for each synthesis (item #5)).	3
	13b	Describe any methods required to prepare the data for presentation or synthesis, such as handling of missing summary statistics, or data conversions.	2-3
	13c	Describe any methods used to tabulate or visually display results of individual studies and syntheses.	2-3
	13d	Describe any methods used to synthesize results and provide a rationale for the choice(s). If meta-analysis was performed, describe the model(s), method(s) to identify the presence and extent of statistical heterogeneity, and software package(s) used.	2-3
	13e	Describe any methods used to explore possible causes of heterogeneity among study results (e.g. subgroup analysis, meta-regression).	2-3
	13f	Describe any sensitivity analyses conducted to assess robustness of the synthesized results.	2-3
Reporting bias assessment	14	Describe any methods used to assess risk of bias due to missing results in a synthesis (arising from reporting biases).	3
Certainty assessment	15	Describe any methods used to assess certainty (or confidence) in the body of evidence for an outcome.	2-3
**RESULTS**			
Study selection	16a	Describe the results of the search and selection process, from the number of records identified in the search to the number of studies included in the review, ideally using a flow diagram.	3-4
	16b	Cite studies that might appear to meet the inclusion criteria, but which were excluded, and explain why they were excluded.	3-4
Study characteristics	17	Cite each included study and present its characteristics.	4
Risk of bias in studies	18	Present assessments of risk of bias for each included study.	8; Suppl material
Results of individual studies	19	For all outcomes, present, for each study: (a) summary statistics for each group (where appropriate) and (b) an effect estimate and its precision (e.g. confidence/credible interval), ideally using structured tables or plots.	4-8
Results of syntheses	20a	For each synthesis, briefly summarise the characteristics and risk of bias among contributing studies.	4-8
	20b	Present results of all statistical syntheses conducted. If meta-analysis was done, present for each the summary estimate and its precision (e.g. confidence/credible interval) and measures of statistical heterogeneity. If comparing groups, describe the direction of the effect.	4-8

Section and Topic	Item #	Checklist Item	Location where Item is Reported
	20c	Present results of all investigations of possible causes of heterogeneity among study results.	4-8; Suppl. material
	20d	Present results of all sensitivity analyses conducted to assess the robustness of the synthesized results.	n/a
Reporting biases	21	Present assessments of risk of bias due to missing results (arising from reporting biases) for each synthesis assessed.	Suppl. material
Certainty of evidence	22	Present assessments of certainty (or confidence) in the body of evidence for each outcome assessed.	8; Suppl. material
**DISCUSSION**			
Discussion	23a	Provide a general interpretation of the results in the context of other evidence.	8-9
	23b	Discuss any limitations of the evidence included in the review.	9
	23c	Discuss any limitations of the review processes used.	9
	23d	Discuss implications of the results for practice, policy, and future research.	9
**OTHER INFORMATION**			
Registration and protocol	24a	Provide registration information for the review, including register name and registration number, or state that the review was not registered.	2
	24b	Indicate where the review protocol can be accessed, or state that a protocol was not prepared.	2
	24c	Describe and explain any amendments to information provided at registration or in the protocol.	2-3
Support	25	Describe sources of financial or non-financial support for the review, and the role of the funders or sponsors in the review.	10
Competing interests	26	Declare any competing interests of review authors.	10
Availability of data, code and other materials	27	Report which of the following are publicly available and where they can be found: template data collection forms; data extracted from included studies; data used for all analyses; analytic code; any other materials used in the review.	n/a

From: Page MJ, McKenzie JE, Bossuyt PM, Boutron I, Hoffmann TC, Mulrow CD, et al. The PRISMA 2020 statement: an updated guideline for reporting systematic reviews. BMJ 2021;372:n71. doi: 10.1136/bmj.n71

For more information, visit: http://www.prisma-statement.org/
